# Hepatocyte-Specific Co-Delivery of Zinc Ions and Plasmid DNA by Lactosylated Poly(1-vinylimidazole) for Suppression of Insulin Receptor Internalization

**DOI:** 10.3390/pharmaceutics13122084

**Published:** 2021-12-04

**Authors:** Akito Endo, Shoichiro Asayama

**Affiliations:** Department of Applied Chemistry, Tokyo Metropolitan University, Hachioji 192-0397, Japan; endo-akito@ed.tmu.ac.jp

**Keywords:** lactosylated poly(1-vinylimidazole), zinc ion, hepatocyte-specific delivery, insulin receptor, hepatic insulin clearance

## Abstract

The lactosylated poly(1-vinylimidazole) (PVIm-Lac) with various lactosylated degrees has been synthesized for the co-delivery of zinc ions (Zn) and plasmid DNA (pDNA). The Zn/DNA/PVIm-Lac complex formation has achieved the specific delivery of zinc ions to HepG2 cells. Especially, the resulting hepatocyte-specific delivery of zinc ions has increased the number of insulin receptors on the cell surface. Consequently, the Zn/DNA/PVIm-Lac complexes have suppressed insulin receptor internalization on the surface of the HepG2 cells, expecting to offer unique therapy to inhibit hepatic insulin clearance.

## 1. Introduction

Zinc ion (Zn^2+^) is known to be an intracellular second messenger, like calcium ion (Ca^2+^), related to various biological functions [1,2,3]. Intracellular free Zn^2+^ ions are considered to be in the range from nM to pM because of the metal-binding affinities of many zinc metalloproteins in that range [4,5,6]. For keeping Zn^2+^ ions at certain concentrations, zinc transporter proteins are responsible [7]. The zinc transporter proteins are divided into two major families, such as ZIP and ZnT, which transport Zn^2+^ ions in opposite directions through cellular and intracellular membranes [8]. ZIPs increase Zn^2+^ concentration in the cytosol to carry Zn^2+^ ions from extracellular and intracellular compartments to the cytosol. Conversely, ZnTs reduce Zn^2+^ concentration in the cytosol to carry Zn^2+^ ions from the cytosol to extracellular and intracellular compartments. Among ZnTs, variants in the human *SLC30A8* gene have been identified through studies in the past decade. The gene encodes the zinc transporter-8 (ZnT8), delivering Zn^2+^ ions from the cytoplasm to the insulin secretory granules in pancreatic β cells [9], affecting the risk of type 2 diabetes [10,11]. Zn^2+^ ions, which were secreted in concert with insulin, suppress hepatic insulin clearance by the inhibition of clathrin-dependent endocytosis of the insulin receptors of hepatocytes in the liver in vivo [12]. We have interpreted that the suppression of the hepatic insulin clearance by Zn^2+^ ions results in the reach of enough insulin to the whole body via blood circulation, leading to type 2 diabetes therapy without insulin injection. In the liver, furthermore, a cooperative function of insulin receptor substrates 1 and 2 are required for insulin signal transduction [13]. Therefore, these studies have inspired us to deliver Zn^2+^ ions to the liver in vivo for hepatic insulin clearance in the viewpoint of type 2 diabetes therapy.

We have previously reported on the design of lactosylated poly(1-vinylimidazole) (PVIm-Lac) for hepatocyte-specific plasmid DNA (pDNA) carriers [14]. Furthermore, we have also reported on Zn^2+^-chelated poly(1-vinylimidazole) (PVIm) derivatives for efficient pDNA delivery [15,16,17,18,19]. These pDNA delivery systems by Zn^2+^-chelated PVIm derivatives have succeeded in the co-delivery of Zn^2+^ ions and pDNA, leading to enhancing the gene expression of the co-delivered pDNA. Especially, the detailed investigation through the use of methylated PVIm (PVIm-Me) has revealed that the biochemical function of the delivered intracellular Zn^2+^ ions, such as activation of the nuclear protein, enhances the nuclear translocation of the co-delivered pDNA [17]. The resulting nuclear translocation induced by the Zn^2+^ ions leads to the up-regulation of the gene expression of the co-delivered pDNA.

Here, to achieve the specific delivery of Zn^2+^ ions to the liver for hepatic insulin clearance, based on our delivery systems, we have used PVIm-Lac as a hepatocyte-specific Zn^2+^ carrier as well as a pDNA carrier. Accordingly, for hepatocyte-specific co-delivery of Zn^2+^ ions and pDNA, we have synthesized PVIm-Lac with various lactosylated degrees to optimize the chemical structure. Subsequently, through the use of each resulting PVIm-Lac, we mainly report the formation ability of the Zn^2+^/pDNA/PVIm-Lac complex, that is, Zn/DNA/PVIm-Lac, followed by the determination of both the number of delivered intracellular Zn^2+^ ions and the number of insulin receptors on the surface of the HepG2 cells derived from human hepatocytes. From the above point of view, because ZnT8 is known to regulate hepatic insulin clearance [12], the optimization of hepatocyte-specific intracellular Zn^2+^ delivery systems are expected to be used for unique type 2 diabetes therapy without insulin injection. Although insulin is secreted from pancreatic β cells, Zn^2+^ ion as a drug is not necessary for delivery to the pancreas (β cell) but instead the liver (hepatocyte). The hepatocyte-specific delivery of Zn^2+^ ions, based on our delivery systems, is considered to achieve no side effects and to offer facile pharmaceutical formulation, so this study is important for type 2 diabetes therapy. 

## 2. Materials and Methods

### 2.1. Materials

Zinc acetate (Zn(OAc)_2_) and branched poly(ethylenimine) (bPEI: 25-kDa) were purchased from Kanto Chemical Co., Inc. (Tokyo, Japan) and Sigma-Aldrich (St. Louis, MO, USA), respectively. All other chemicals were of a special grade and used without further purification. 

### 2.2. Synthesis of PVIm-Lac with Various Lactosylated Degrees

Lactosylated poly(1-vinylimidazole), PVIm-Lac, was generally synthesized according to our previous paper [14]. In this study, three kinds of PVIm-Lac with a higher lactosylated degree than our previous study were synthesized as follows: the PVIm (200 mg: number-average molecular weight of 7.8 × 10^3^) and 93, 232 or 348 mg of 3-bromopropylamine were dissolved in 7 mL of dimethyl sulfoxide (DMSO), followed by incubation at 40 °C for 48 h. Then, to remove unreacted 3-bromopropylamine, we carried out the dialysis of the resulting mixtures against distilled water using a Spectra/Por 7 membrane (REPLIGEN, Waltham, MA, USA) (molecular weight cutoff = 3500). After the dialysis, aminopropylated poly(1-vinylimidazole), PVIm-NH_2_, was obtained by freeze-drying.

Subsequently, PVIm-NH_2_ (corresponding to 0.1 mmol of amino groups) and the lactose monohydrate (144 mg: 0.4 mmol) were preincubated at 37 °C for 72 h in 1.75 mL of sodium borate buffer (0.1 M, pH 8.5), followed by further incubation with a reducing agent, NaBH_3_CN, at 37 °C for 72 h in the final volume of 3 mL of sodium borate buffer (0.1 M, pH 8.5). The primary amino group determination with fluorescamine [20] was carried out to examine the degree of amino group modification with lactose. After the determination, to remove unreacted lactose, we examined the dialyzed substance against distilled water using a Spectra/Por 7 membrane (molecular weight cutoff = 3500). After the dialysis, a PVIm-Lac with various lactosylated degrees was obtained by freeze-drying.

### 2.3. Gel Filtration Chromatography (GFC)

GFC was performed by a JASCO PU-980 pumping system (Tokyo, Japan) with a Shodex OHpak SB-804 HQ column (Showa Denko K. K., Tokyo, Japan) at a flow rate of 1.0 mL/min. The mobile phase containing CH_3_COOH (0.5 M) and NaNO_3_ (0.2 M) was used. In the column, 100 μL of 1 mg/mL samples were injected. The eluate was detected with an RI detector (RI-1530; JASCO, Tokyo, Japan). Polyethylene glycol standards were used for calibration.

### 2.4. ^1^H NMR Spectroscopy

The ^1^H NMR spectra (500 MHz), in 500 μL of D_2_O (99.8 atom % deuterium; Kanto Chemical, Japan) dissolving the resulting polymer (5 mg), were measured by a Bruker AV500 spectrometer (Billerica, MA, USA).

### 2.5. Agarose Gel Retardation Assay

The mixture of a stock solution of each PVIm-Lac and the dilution of pDNA stock solution (300 ng of pDNA) with physiological saline (150 mM NaCl), adjusted to 13.5 μL at various positive/negative charge ratios ([Imidazolium and aminopropyl]_PVIm-Lac_/[Phosphate]_pDNA_), was incubated at 37 °C for 1 h. The resulting sample mixed with a 1.5 μL of loading buffer was loaded onto a 1% agarose gel in the presence of ethidium bromide (1 μg/mL). In TAE buffer (Tris-acetate, EDTA), gel electrophoresis was performed at room temperature (50 V, 30 min), followed by the visualization of the pDNA bands under UV irradiation. 

For the Zn/DNA/PVIm-Lac complex formation assay, after preincubation of each PVIm-Lac with 1.5 mM zinc acetate (Zn(OAc)_2_) at 37 °C for 1 h, the above electrophoresis experiments were carried out. In the case of the complex stability assay, the above agarose gel electrophoresis was run after the incubation of each sample at 37 °C for 10 min in the presence of dextran sulfate (DS: 2mM as sulfate group).

### 2.6. Particle Size and ζ-Potential Measurement

Dynamic light scattering (DLS) determined the size of each Zn/DNA/PVIm-Lac complex (pDNA: 4.5 μg per sample) at a positive/negative charge ratio of 8 in physiological saline, as well as corresponding controls, by an electrophoresis light scattering spectrophotometer (ELS-Z2, Otsuka Electronics Co., Ltd., Tokyo, Japan). Moreover, the ζ-potential of the resulting sample was measured by the ELS with electrodes in physiological saline.

### 2.7. Cell Viability Assay

Human hepatoma cell line HepG2 cells (from Riken Bioresource Center Cell Bank, Ibaraki, Japan), as a representative cell, were cultured in tissue culture flasks in Dulbecco’s modified Eagle’s medium supplemented with 10% heat-inactivated FBS. The cells were seeded at 1×10^4^ cells/well in a 96-well plate (100 μL/well) and incubated overnight at 37 °C in a 5% CO_2_ incubator. Then, the Zn/DNA/PVIm-Lac complexes (15 μL/well, pDNA: 300 ng per sample as Section 2.5) were added to the cells and incubated for 24 h at 37 °C. After additional incubation for 4 h, the cell viability assay was performed by the Alamar Blue assay [21] in triplicate.

### 2.8. Cellular Uptake of Zn^2+^ Ions

As above in Section 2.7, HepG2 cells and human cervical cancer cell line HeLa cells (from Riken Bioresource Center Cell Bank) were incubated for 24 h and 37 °C with the Zn/DNA/PVIm-Lac complexes (15 μL/well, pDNA: 300 ng per sample as Section 2.5). After the incubation and washing of the cells with PBS (+), 20 μL of 1% Triton X-100 and 80 μL of H_2_O were added to the cells. Subsequently, 100 μL of the resulting cell lysate was diluted to 1.4 mL with H_2_O containing hydrochloric acid. The atomic absorption spectrometry at 213.9 nm determined the concentration of Zn^2+^ in the resulting sample by the atomic absorption spectrophotometer AA-6200 (Shimazu Co., Kyoto, Japan). 

### 2.9. Transfection Procedure

As above Section 2.8, HepG2 cells and HeLa cells were transfected by the Zn/DNA/PVIm-Lac complexes (15 μL/well, pDNA: 300 ng per sample as Section 2.5). Each pDNA encoding the modified firefly luciferase was used, and bPEI was used as a positive control. After 1 day of incubation, the medium was removed, and the cells were further incubated for 2 days. For the competitive inhibition assay of the asialoglycoprotein receptor binding, the transfection experiments were carried out in the presence of excess lactose molecules. The cells were washed with PBS (−) twice. Then, according to the manufacturer’s instructions, the luciferase assay (Promega kit) was performed. Luciferase activities were normalized to protein concentrations and presented as relative light units (RLU). For the determination of the protein concentrations, the bicinchonic acid (BCA) protein assay (Pierce) was performed according to the manufacturer’s instructions.

### 2.10. Insulin Receptor Cell-Based Enzyme-Linked Immunosorbent Assay (ELISA)

As above in Section 2.9, HepG2 cells were transfected by the Zn/DNA/PVIm-Lac complexes (15 μL/well, pDNA: 300 ng per sample as Section 2.5). After 1 day of incubation, the insulin receptors (INSRs) on the cell surface were detected with INSR (Human) cell-based ELISA kit (Abnova, Taipei, Taiwan), according to the manufacturer’s instructions. 

### 2.11. Statistical Analysis

Statistical analysis was performed using a Student’s *t*-test. 

## 3. Results and Discussion

### 3.1. Synthesis of PVIm-Lac with Various Lactosylated Degrees

**Figure 1:**
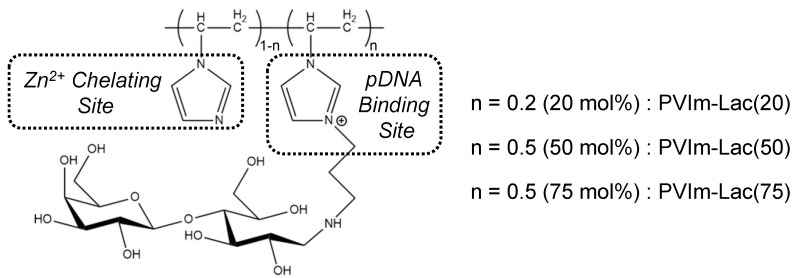
Chemical structure of PVIm-Lac with various lactosylated degrees.

Figure 1 shows the chemical structure of PVIm-Lac with various lactosylated degrees. The number-average molecular weight of the PVIm backbone was approximately 7.8 × 10^3^, estimated by GFC (Figure S1). The modification degree of aminopropylated imidazole groups of each PVIm-NH_2_ was determined to be 20, 50, or 75 mol% from the signal ratio of each ^1^H NMR spectrum (Figure S2). After the subsequent conjugation of PVIm-NH_2_ with lactose molecules, early elution shift and no free lactose were observed in GFC chromatograms (Figure S1). Furthermore, ^1^H NMR spectra indicated the characteristic signals of both the PVIm-NH_2_ backbone and lactose (Figure S2). Through the primary amino group determination using fluorescamine [20], almost no free amino groups were detected after the conjugation of PVIm-NH_2_ with lactose (Figure S3). These results suggest that the amino groups of PVIm-NH_2_ were almost completely modified with lactose molecules. Thus, we have synthesized the lactosylated PVIm modified with 20, 50, or 75 mol% lactosylated degrees, that is, PVIm-Lac(20), PVIm-Lac(50), or PVIm-Lac(75).

### 3.2. Formation of Zn/DNA/PVIm-Lac Complexes

**Figure 2:**
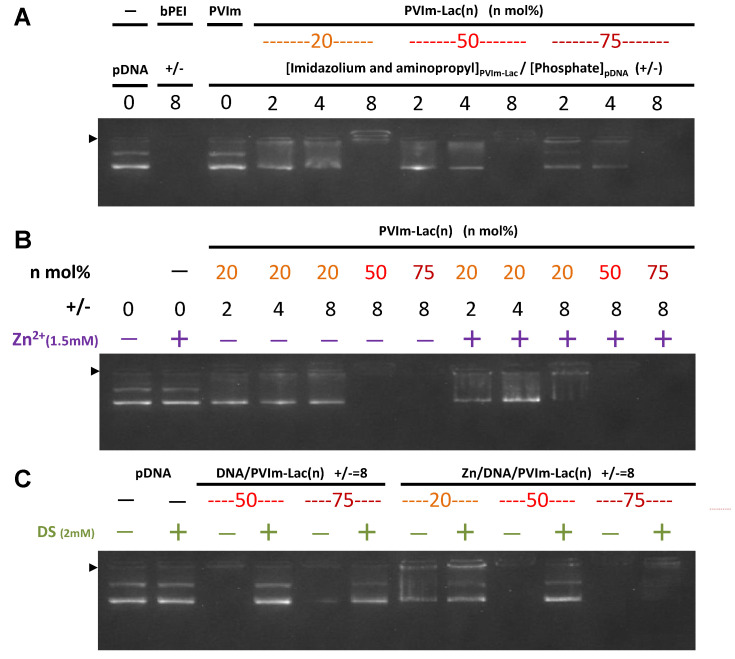
(**A**) Analysis of the DNA/PVIm-Lac PIC formation by agarose gel electrophoresis. The lactosylated degree (*n*: mol%) and positive/negative (+/−) mixing charge ratios are indicated. As a control, bPEI was used. The solid arrowhead indicated the well where each sample was loaded. (**B**) Analysis of the Zn/DNA/PVIm-Lac complex formation. The mixtures of PVIm-Lac and pDNA were loaded in the absence (−) or presence (+) of Zn^2+^ ions (1.5 mM). (**C**) Release of pDNA from the Zn/DNA/PVIm-Lac complexes by dextran sulfate (DS +: 2 mM).

As shown in Figure 2A, we first examined the polyion complex (PIC) formation between PVIm-Lac and pDNA by agarose gel electrophoresis. As a positive control, pDNA/bPEI complexes at a positive/negative charge ratio of eight exhibited no fluorescence from ethidium bromide staining because the coil–globule transition of the pDNA inhibited ethidium bromide intercalation [22,23]. In the case of all PVIm-Lac, the complete retardation of the pDNA was observed at a positive/negative charge ratio of eight, suggesting the complete formation of DNA/PVIm-Lac PICs. Looking in detail, the resulting retarded band of PVIm-Lac(75) exhibited no fluorescence (max. 73 and min. 42 in the quantified area), whereas that of PVIm-Lac(20) exhibited a little fluorescence (max. 142 and min. 57 in the quantified area). Therefore, because of a higher cationic density, PVIm-Lac with a higher lactosylated degree is considered to induce more coil–globule transition of anionic pDNA.

Subsequently, as shown in Figure 2B, we examined the Zn/DNA/PVIm-Lac complex formation. In the presence of Zn^2+^ ions, no retardation of the naked pDNA (+/− = 0) was observed, suggesting no complex formation between the Zn^2+^ ions and pDNA under these experimental conditions. Conversely, more retardation of the pDNA was especially observed in the case of PVIm-Lac(20) at a positive/negative charge ratio of eight in the presence of Zn^2+^ ions, as compared to the absence of Zn^2+^ ions. These results suggest that the chelated Zn^2+^ ions with PVIm-Lac(20), as well as PVIm-Lac(50) and PVIm-Lac(75), worked as a polycation. Therefore, the Zn/DNA/PVIm-Lac complexes are considered to form. The particle size and ζ-potential of the resulting Zn/DNA/PVIm-Lac complexes were approximately 100–120 nm and +2–5 mV, respectively (Table S1), suggesting no surplus cross-linking and aggregation.

Furthermore, as shown in Figure 2C, the stability of the resulting Zn/DNA/PVIm-Lac complexes was examined in the presence of dextran sulfate (DS). For the DNA/PVIm-Lac PICs, the migration of pDNA bands was observed, suggesting the release of pDNA from the DNA/PVIm-Lac PICs through competitive exchange with the DS. Especially for Zn/DNA/PVIm-Lac(75), on the other hand, no migration of the pDNA bands was observed. These results suggest that the Zn^2+^ chelation to imidazole groups of PVIm-Lac(75) retained pDNA more tightly, as compared to the DNA/PVIm-Lac(75) PIC. 

### 3.3. Cell Viability in the Presence of Zn/DNA/PVIm-Lac Complexes

**Figure 3:**
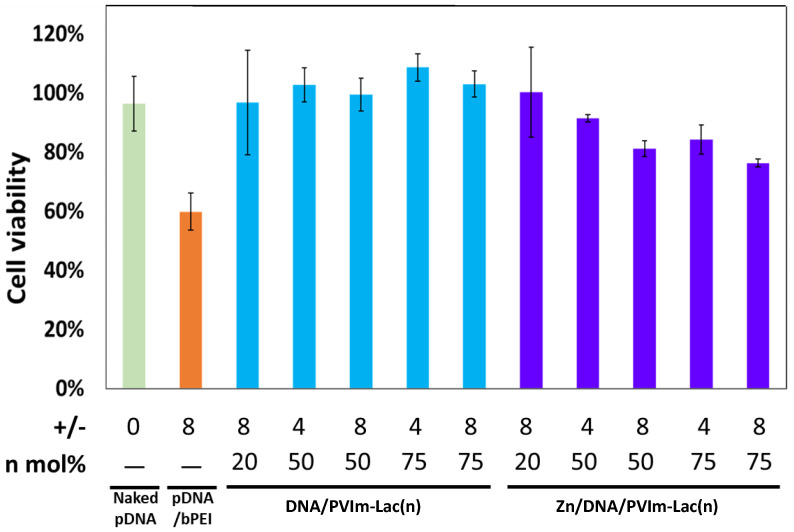
Cell viability in the presence of Zn/DNA/PVIm-Lac complexes as well as DNA/PVIm-Lac PICs. HepG2 cells were used for the assay. The lactosylated degree (*n*: mol%) and positive/negative (+/−) mixing charge ratios are represented. A control, bPEI, was used. Data and error bars represent the mean and standard deviation (*n* = 3).

We examined the human hepatoma HepG2’s cell viability in the presence of Zn/DNA/PVIm-Lac complexes because no cytotoxicity is desirable for the co-delivery of Zn^2+^ ions and pDNA with each PVIm-Lac. As shown in Figure 3, although cell viability was decreased up to 60% in the presence of pDNA/bPEI PIC, almost 100% cell viability was observed in the presence of the DNA/PVIm-Lac complexes, proving no cytotoxicity of each PVIm-Lac. Similarly, in the presence of the Zn/DNA/PVIm-Lac complexes, almost 80% cell viability was maintained. In particular, the resulting 80% cell viability, in the presence of the Zn/DNA/PVIm-Lac(50) or Zn/DNA/PVIm-Lac(75) complex at a positive/negative charge ratio of eight, is not statically significant (*p* > 0.1) against the naked pDNA (100% cell viability). Therefore, these results suggest there is negligible cytotoxicity of Zn^2+^ ions in the complexes. 

### 3.4. Intracellular Delivery of Zn^2+^ Ions by Zn/DNA/PVIm-Lac Complexes

**Figure 4:**
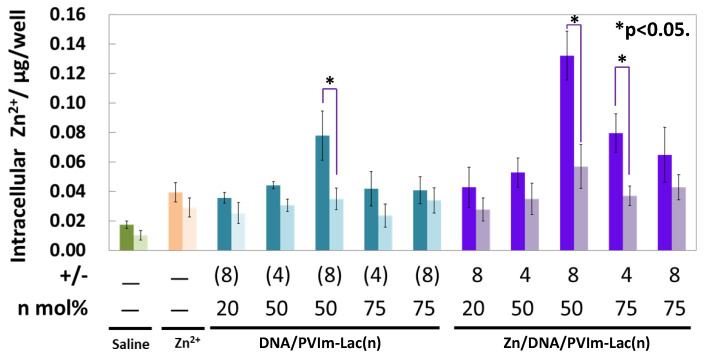
Cellular uptake of Zn^2+^ ions by Zn/DNA/PVIm-Lac complexes as well as Zn/PVIm-Lac complexes. HepG2 (left bar) or HeLa (right bar) cells were used for the assay. The lactosylated degree (*n*: mol%) and positive/negative (+/−) mixing charge ratios are represented. Data and error bars represent the mean and standard deviation (*n* = 3). There is statistical significance (* *p* < 0.05) between HepG2 cells and HeLa cells.

As shown in Figure 4, the intracellular delivery of Zn^2+^ ions through the Zn/DNA/PVIm-Lac complexes was examined because of no significant cytotoxicity in the Zn/DNA/PVIm-Lac complexes. In the case of delivery into the HepG2 cells, the highest amount of Zn^2+^ ions was determined by the use of the Zn/DNA/PVIm-Lac(50) complex at a positive/negative charge ratio of eight. Because the amount of Zn^2+^ ions determined by use of the Zn/DNA/PVIm-Lac(50) complex was higher than that by the use of the Zn/PVIm-Lac(50) complex (without pDNA), the DNA/PVIm-Lac(50) PIC formation may enhance Zn^2+^ chelation, due to a formed hydrophobic microenvironment around imidazole groups of PVIm-Lac(50) [18]. Notably, the number of Zn^2+^ ions inside human cervical cancer HeLa cells without asialoglycoprotein receptors was statistically significantly lower than that inside the HepG2 cells (*p* < 0.05). These results suggest that the Zn/DNA/PVIm-Lac(50) complexes with β-galactose residues were a suitable structure for cell-specific Zn^2+^ delivery to recognize the HepG2 cells via asialoglycoprotein receptors on the cell surface [16].

### 3.5. Gene Expression of pDNA Delivered by Zn/DNA/PVIm-Lac Complexes

**Figure 5:**
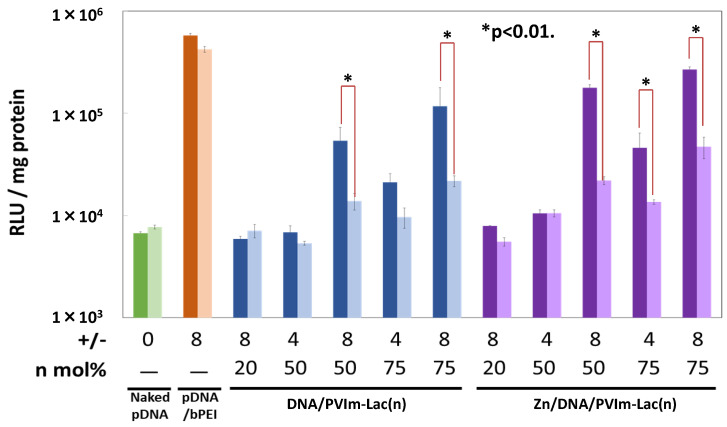
Transfection of luciferase gene into HepG2 (left bar) or HeLa (right bar) cells by Zn/DNA/PVIm-Lac complexes as well as DNA/PVIm-Lac PICs. As a positive control, bPEI was used. The lactosylated degree (*n*: mol%) and positive/negative (+/−) mixing charge ratios are indicated. Gene expression was determined as RLU normalized by protein concentrations. Data and error bars represent the mean and standard deviation (*n* = 3). There is statistical significance (* *p* < 0.01) between HepG2 cells and HeLa cells.

As a result of cell-specific Zn^2+^ delivery, we examined the pDNA gene expression mediated by the Zn/DNA/PVIm-Lac complexes. As shown in Figure 5, the PVIm-Lac(75) with the highest lactosylated degree, as well as a higher positive/negative charge ratio of eight, mediated the highest expression of the pDNA inside the HepG2 cells in the presence of Zn^2+^ ions. Generally, PVIm-Lac(50) mediated the pDNA expression, whereas PVIm-Lac(20) did not mediate the expression. Furthermore, the pDNA expression in the presence of Zn^2+^ ions was higher than that in the absence of Zn^2+^ ions. These results suggest that the tightest retention of the pDNA in the Zn/DNA/PVIm-Lac(75) complexes (Figure 2C) worked advantageously for the pDNA delivery inside the HepG2 cells. Furthermore, Zn^2+^ ions are considered to up-regulate gene expression of the co-delivered pDNA [17]. 

It should be noted that the resulting pDNA expression of the HeLa cells was statistically significantly lower than that of the HepG2 cells (* *p* < 0.01). The lower gene expression of the HeLa cells is considered to be almost the same tendency as the lower amount of Zn^2+^ ions inside the HeLa cells (Figure 4). Furthermore, in the presence of excess lactose molecules for the competitive inhibition assay of the asialoglycoprotein receptor binding with each PVIm-Lac, the resulting gene expression of the HepG2 cells decreased, as compared to that in the absence of lactose molecules (Figure S4). These results suggest that the Zn/DNA/PVIm-Lac complexes, as well as the DNA/PVIm-Lac PICs, succeeded in the cell-specific pDNA delivery to recognize the HepG2 cells via asialoglycoprotein receptors on the cell surface through the use of PVIm-Lac(50) or PVIm-Lac(75) with more β-galactose residues, as compared to PVIm-Lac(25).

### 3.6. Suppression of Insulin Receptor Internalization by Zn/DNA/PVIm-Lac Complexes

**Figure 6:**
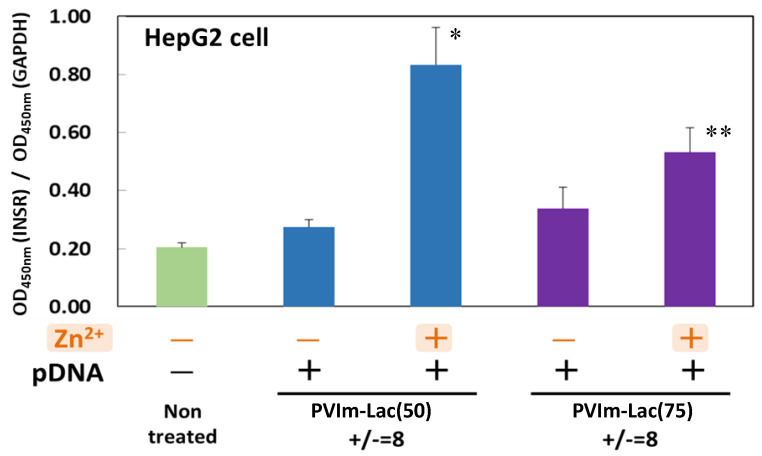
Internalization assay for insulin receptors on the surface of HepG2 cells. HepG2 cells were treated with Zn/DNA/PVIm-Lac complexes, followed by the determination of cell surface insulin receptor (INSR). The target absorbance value (OD_450nm_) is normalized by an internal positive control, glyceraldehyde-3-phosphate dehydrogenase (GAPDH). The lactosylated degree (*n*: mol%) and positive/negative (+/−) mixing charge ratios are indicated. Data and error bars represent the mean and standard deviation (*n* = 3). There is statistical significance (* *p* < 0.005, ** *p* < 0.01), as compared to non-treated cells.

Based on the cell-specific co-delivery of the Zn^2+^ ions and pDNA for the HepG2 cells from human hepatocytes through the use of PVIm-Lac(50) or PVIm-Lac(75), as shown in Figure 6, we finally examined whether these Zn/DNA/PVIm-Lac complexes suppressed the internalization of insulin receptors on the cell surface. When the HepG2 cells were treated with the DNA/PVIm-Lac PICs, the number of insulin receptors on the cell surface was the almost same level as that of non-treated cells. It is worth noting that treatment of the HepG2 cells with the Zn/DNA/PVIm-Lac complexes significantly increased the number of insulin receptors on the cell surface as compared to that of the non-treated cells (* *p* < 0.005, ** *p* < 0.01). The number of insulin receptors on the HepG2 cell surface treated with the Zn/DNA/PVIm-Lac(50) complex was higher than that treated with the Zn/DNA/PVIm-Lac(75) complex. The intracellular amount of Zn^2+^ ions delivered by the Zn/DNA/PVIm-Lac(50) complex was higher than that delivered by the Zn/DNA/PVIm-Lac(75) complex (Figure 4), so that the number of cell surface insulin receptors is considered to be related to the intracellular number of Zn^2+^ ions. 

Because the decrease in cell-surface insulin receptors is reported to be suppressed by co-treatment of Zn^2+^ ions with HepG2 cells [12], the increase in the intracellular amount of Zn^2+^ ions delivered by the Zn/DNA/PVIm-Lac complexes suggests suppressing the internalization of cell-surface insulin receptors. Furthermore, the internalization of insulin is also reported to be inhibited by Zn^2+^ ions [12], according to the suppression of the internalization of cell-surface insulin receptors. Our preliminary functional study with insulin kinetics also supports the theory that the internalization of insulin is inhibited by the delivery of Zn^2+^ ions inside HepG2 cells, resulting in more residual insulin in the medium, as compared to that without the delivery of Zn^2+^ ions (Figure S5). If intravenous (i.v.) administration of Zn/DNA/PVIm-Lac complexes delivers Zn^2+^ ions to the liver, therefore, the residual insulin without hepatic insulin clearance is considered to reach the whole body via blood circulation, affecting the glucose kinetics of the target cells in the whole body. Taking these results into account, although only in vitro (in cells) experiments were carried out in this study, the delivery of Zn^2+^ ions to the liver in vivo (in whole organisms) by the Zn/DNA/PVIm-Lac complexes is expected to inhibit hepatic insulin clearance during the first liver passage for the unique diabetes therapy without insulin injection. Due to there being no side effects, based on the specificity of HepG2 cells compared to HeLa cells, the hepatocyte-specific delivery of Zn^2+^ ions in this study is considered to be safe for in vivo administration.

## 4. Conclusions

In summary, we have synthesized lactosylated poly(1-vinylimidazole), that is, PVIm-Lac, with various lactosylated degrees. Each PVIm-Lac has formed the polyion complex with the Zn^2+^ ions and pDNA. The resulting Zn/DNA/PVIm-Lac complexes have exhibited negligible cytotoxicity for co-delivery of Zn^2+^ ions and pDNA. In particular, the PVIm-Lac with 50 mol% lactosylated degrees, that is, PVIm-Lac(50), has delivered the highest amount of Zn^2+^ ions inside the HepG2 cells from human hepatocytes, and not the HeLa cells from human cervical cancer. Namely, the Zn/DNA/PVIm-Lac(50) complex has achieved the hepatocyte-specific co-delivery of Zn^2+^ ions and pDNA. Notably, the resulting hepatocyte-specific co-delivery of Zn^2+^ ions and pDNA by the Zn/DNA/PVIm-Lac(50) complex has increased the number of insulin receptors on the cell surface. Consequently, the Zn/DNA/PVIm-Lac(50) complex has suppressed insulin receptor internalization on the hepatocyte surface, expecting to offer unique therapy to inhibit hepatic insulin clearance. 

## Data Availability

Data is contained within the article or Supplementary Materials.

## Supplementary Materials

The following are available online at https://www.mdpi.com/article/10.3390/pharmaceutics13122084/s1, Figure S1: GFC chromatograms, Figure S2: ^1^H NMR spectra, Figure S3: Primary amino group determination of PVIm-Lac, Figure S4: Transfection of luciferase gene into HepG2 cells by Zn/DNA/PVIm-Lac complexes in the presence of excess lactose molecules, Figure S5: Determination of insulin in the medium where HepG2 cells were treated with Zn/DNA/PVIm-R complexes, Table S1: Particle size and ζ-potential of the Zn/DNA/PVIm-Lac complexes.
