# Supplementary material for: Hepatocyte-Specific Co-Delivery of Zinc Ions and Plasmid DNA by Lactosylated Poly(1-vinylimidazole) for Suppression of Insulin Receptor Internalization
Source: Pharmaceutics. 2021 Dec 4;13(12):2084. doi: 10.3390/pharmaceutics13122084 (PMC8704993; doi:10.3390/pharmaceutics13122084)
Supplement: Supplementary file 1 [file pharmaceutics-13-02084-s001.zip › pharmaceutics-1453937-supplementary.pdf]

pharmaceutics

Supplementary Materials: Hepatocyte-Specific Co-Delivery of Zinc Ions and Plasmid DNA by Lactosylated Poly(1-vinylimidazole) for Suppression of Insulin Receptor Internalization

Akito Endo and Shoichiro Asayama *

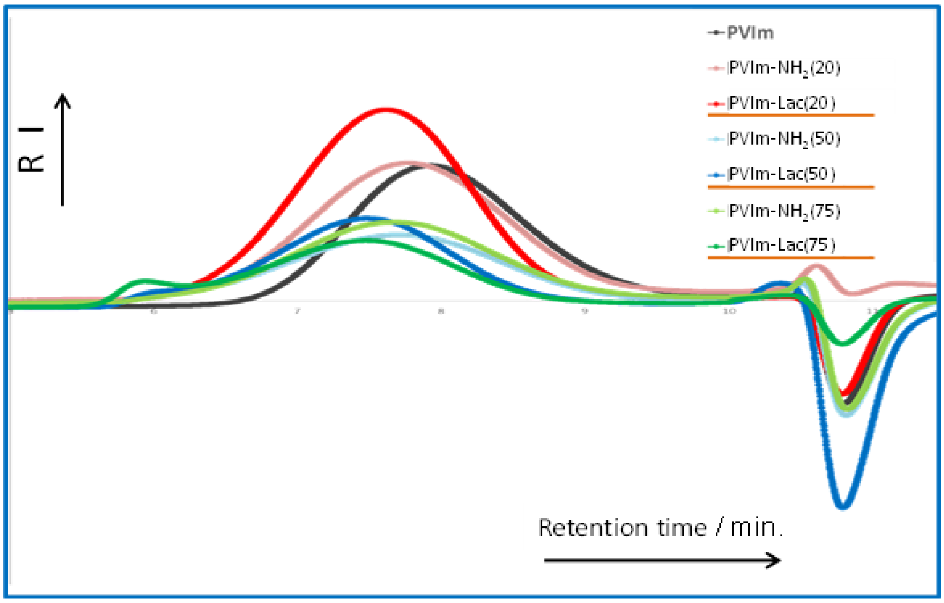

Sample name	Rt
PVIm	7.92
PVIm-NH2 (20)	7.78
PVIm-Lac (20)	7.61
PVIm-NH2 (50)	7.70
PVIm-Lac (50)	7.47
PVIm-NH2 (75)	7.68
PVIm-Lac (75)	7.47

Figure S1. GFC chromatograms of PVIm-NH_2 and PVIm-Lac. RI and Rt means refractive index and retention time, respectively.

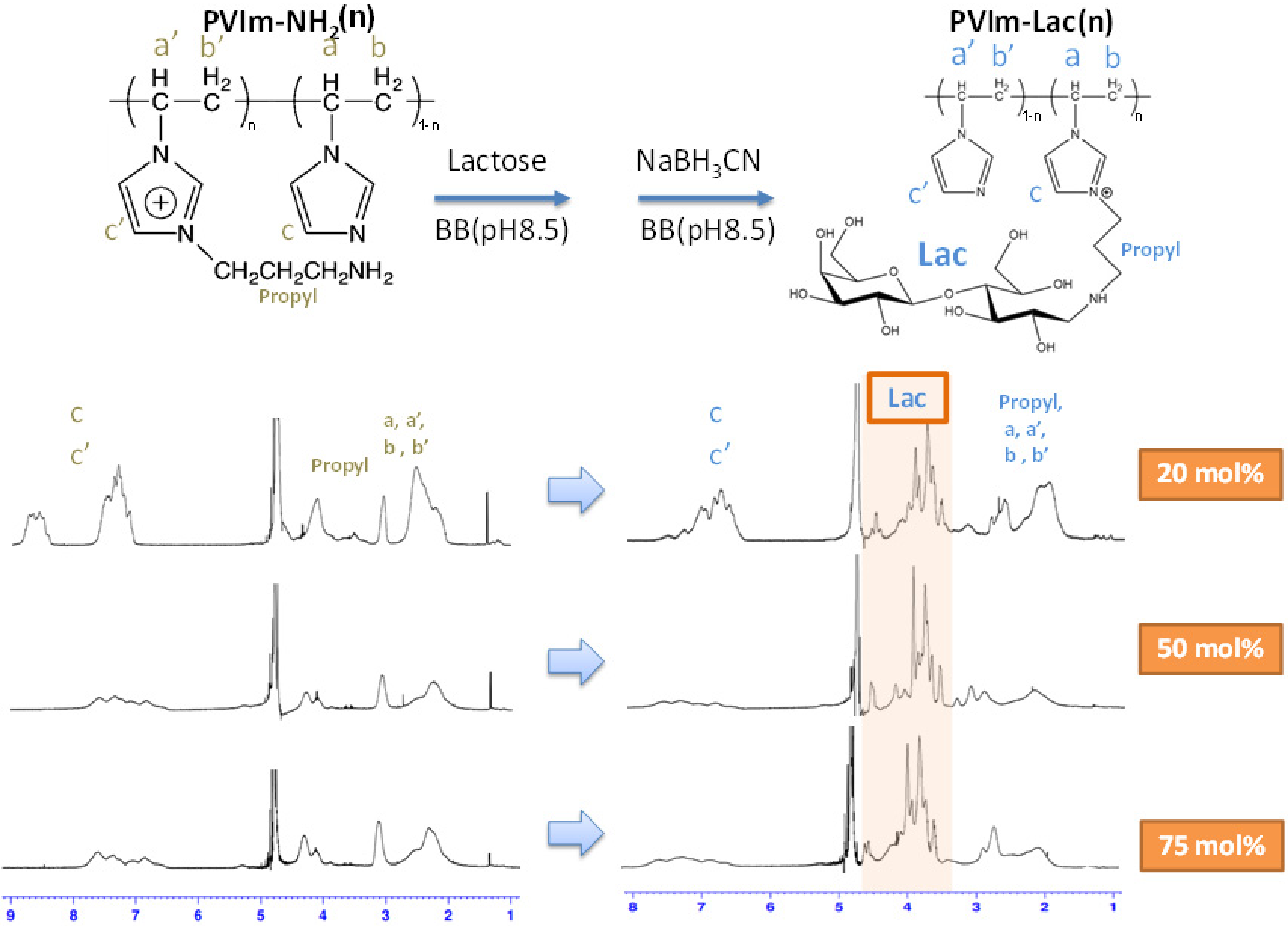

Figure S2. ^{1}H NMR spectra of PVIm-NH_2 and PVIm-Lac in D_2O. BB means sodium borate buffer (0.1 M).

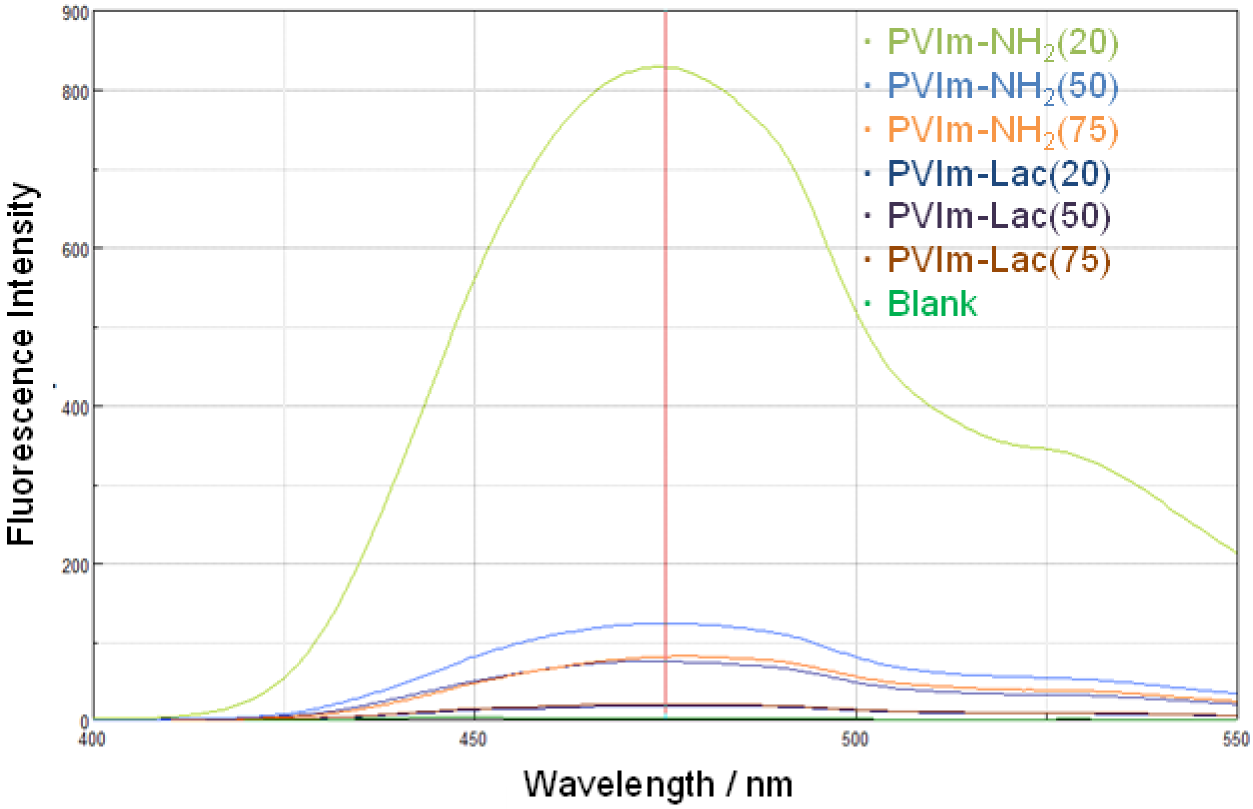

Figure S3. Primary amino group determination, by use of fluorescamine, before (PVIm-NH2) and after (PVIm-Lac) the conjugation with lactose molecules.

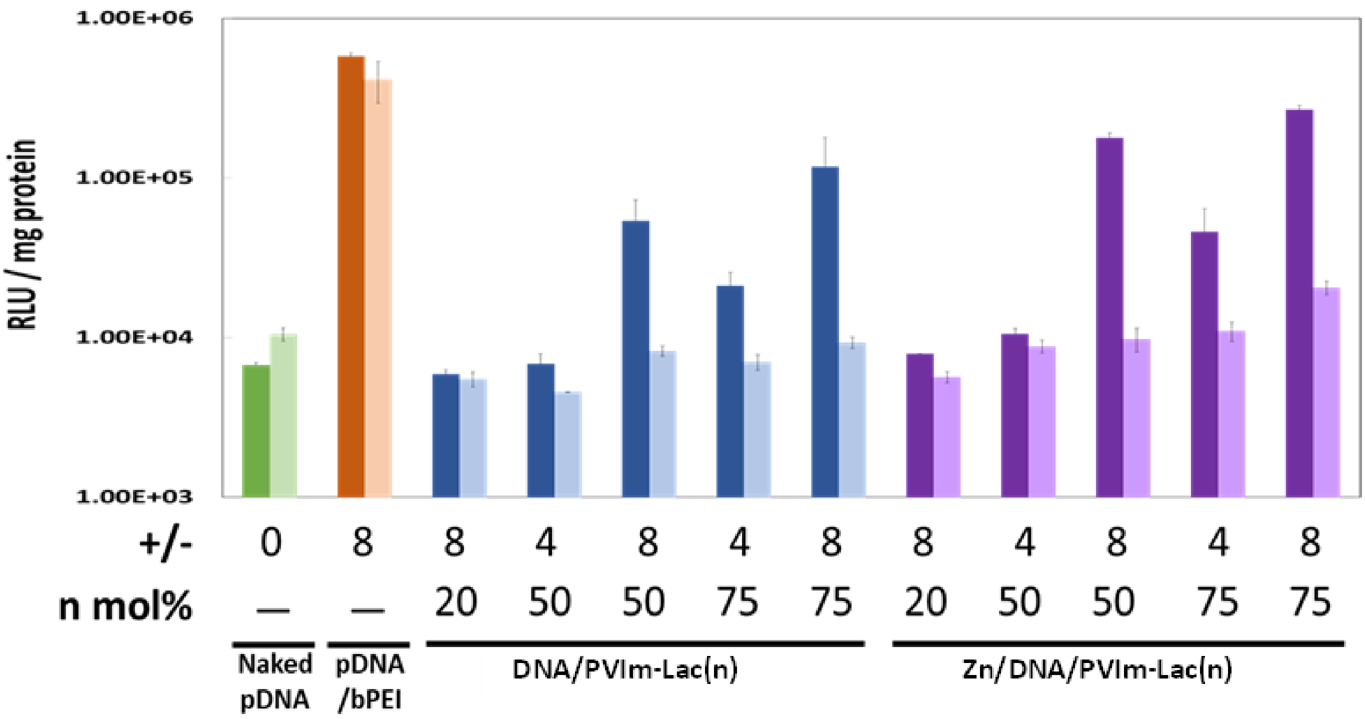

Figure S4. Transfection of luciferase gene into HepG2cells by Zn/DNA/PVIm-Lac complexes as well as DNA/PVIm-Lac PICs in the presence (right bar) of absence (left bar) of excess lactose molecules. As a positive control, bPEI was used. The lactosylated degree (n : mol%) and positive/negative (+/-)

mixing charge ratios are indicated. Gene expression was determined as RLU normalized by protein concentrations. Symbols and error bars represent the mean and standard deviation (*n* = 3).

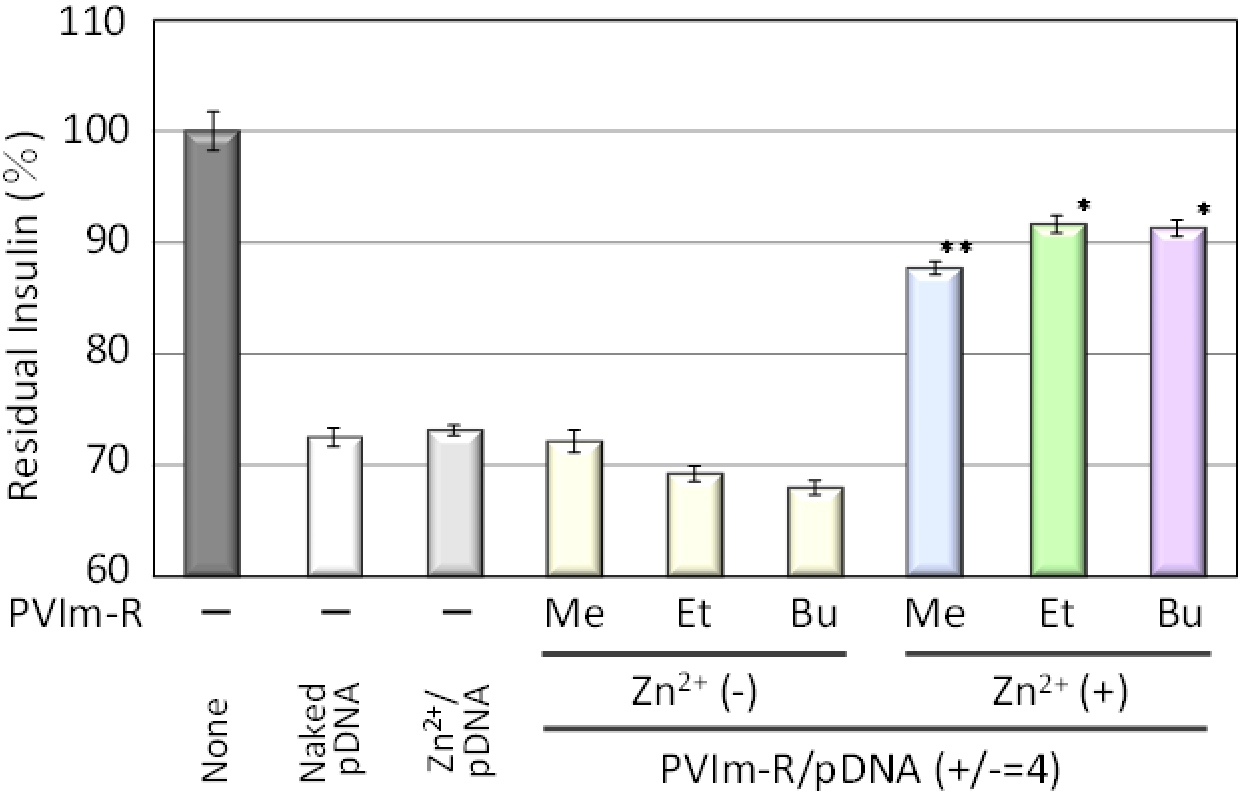

Figure S5. Determination of insulin in the medium where HepG2 cells were treated with the complex (PVIm-R/DNA, +/-=4) between pDNA and each 20 mol% alkylated PVIm (PVIm-R: Me, methyl; Et, ethyl; Bu, butyl) in the presence (+) or absence (-) of Zn^{2+} ions. Residual insulin (%) in the medium was determined by Mercodia ultrasensitive insulin ELISA kit (Uppsala, Sweden), according to the manufacture's protocol. Symbols and error bars represent the mean and standard deviation (*n* = 3). There is statistical significance ($^*p < 0.01$, $^{**}p < 0.05$) between PVIm-R/pDNA complexes (Zn^{2+}(-)) and its corresponding Zn/DNA/PVIm-R complexes (Zn^{2+}(+)).

Table S1. Particle size and ζ-potential of the Zn/DNA/PVIm-Lac complexes.

Sample	Diameter /nm	Zeta potential /mV
**Zn^{2+} alone**	—	—
**PVIm-Lac(50), (75) alone**	—	—
**DNA/PVIm-Lac(50) +/-=8**	123.2±35.5	16.13
**Zn/DNA/PVIm-Lac(50) +/-=8**	128.8±38.8	1.93
**DNA/PVIm-Lac(75) +/-=8**	123.4±30.7	0.38
**Zn/DNA/PVIm-Lac(75) +/-=8**	96.4±23.6	4.57
